# Fruits and vegetable consumption, and its association with hypertension among women in Ghana: a cross-sectional study

**DOI:** 10.1017/S1368980023002896

**Published:** 2023-12-27

**Authors:** Joshua Okyere, Castro Ayebeng, Bernard Afriyie Owusu, Kwamena Sekyi Dickson

**Affiliations:** 1 Department of Population and Health, University of Cape Coast, Cape Coast, University Post Office, PMB, Ghana; 2 Department of Nursing, College of Health Sciences, Kwame Nkrumah University of Science and Technology, Kumasi, Ghana

**Keywords:** Fruits and vegetable consumption, hypertension, public health, Demographic and Health Survey

## Abstract

**Objective:**

This study aimed to examine the association between fruit and vegetable consumption (FVC) and the risk of hypertension among women in Ghana.

**Design:**

Data from the 2014 Ghana Demographic and Health Survey were used. Bivariate and multivariate logistic regression analyses were performed using Stata version 14. The study reports the adjusted OR (AOR) and CI.

**Setting:**

Ghana

**Participants:**

A total sample of 4168 women was used in the analysis.

**Results:**

Among women who met the WHO’s recommended intake of FVC, 13·1 % had hypertension. The intake of the recommended servings of fruit and vegetables was not significantly associated with hypertension. However, the likelihood of being hypertensive was significantly associated with increasing age (AOR = 6·1; 95 % CI = 4·29, 8·73), being married (AOR = 1·7; 95 % CI = 1·14, 2·57) or formerly married (AOR = 2·3; 95 % CI = 1·44, 3·70), and being overweight (AOR = 1·6; 95 % CI = 1·24, 2·07) or obese (AOR = 2·4; 95 % CI = 1·82, 3·20).

**Conclusion:**

The study concludes that there is no significant association between FVC and hypertension risk among women in Ghana. While this study did not find a significant association between FVC and hypertension risk among women in Ghana, it underscores the point that other multifaceted factors influence hypertension risk. As such, public health campaigns should emphasise a balanced and holistic approach to promoting cardiovascular health, including factors beyond FVC. The findings also highlight the need to target high-risk populations (i.e. older women, married and formerly married women, and overweight or obese women) with hypertension prevention education and related interventions.

Globally, hypertension is a leading risk factor for CVD and renal diseases^(1)^. Estimates from the WHO indicate that there are about 1·28 billion adults living with hypertension^(2)^. Two-thirds of this number reside in low- and middle-income countries which includes Ghana^(2)^. In Ghana, the prevalence of hypertension is estimated to be 13·0 % in the general population; however, it differs for males (12·1 %) and females (13·4 %)^(3)^. Moreover, evidence suggests that the prevalence of hypertension in Ghana is higher in the southern parts (30·7 %) of the country as compared to the northern belt (22·9 %)^(4)^.

Hypertension has over the years gained the accolade of being the ‘silent killer’, as it is the major cause of premature deaths^(2)^. Not only does hypertension exacerbate the risk of premature deaths, but also it increases the risk of myocardial infarctions, renal failures and neurological disorders such as strokes^(5,6)^. In addition, the quality of life of people with hypertension is significantly reduced^(7)^, thus, making hypertension a critical public health concern. Therefore, taking preventive measures against hypertension could be instrumental in reducing the likelihood of developing complications such as strokes, CVD and kidney damage^(2)^.

Previous studies have documented the significant association between modifying lifestyles and the risk of developing hypertension. These modifying lifestyles include physical inactivity or sedentary lifestyle, alcohol consumption, red meat consumption and smoking^(8–10)^. Another modifiable lifestyle associated with the risk of hypertension is fruit and vegetable consumption (FVC)^(11)^. According to the WHO^(12)^, a healthy diet should include ‘at least 400 g (i.e. five portions) of fruit and vegetables per d (2), excluding potatoes, sweet potatoes, cassava and other starchy roots’. It is noteworthy that fruits and vegetables contain vitamins, minerals, and folic acid that have been reported to significantly improve endothelium function^(13,14)^. Given the protective factor of fruits and vegetables against endothelial dysfunction, a known risk factor of hypertension^(15)^, it would likely be associated with reduced risk of hypertension.

Consequently, some studies have been conducted to investigate the association between FVC and the risk of hypertension; however, there appear to be inconclusive reports on this hypothesis. While some studies have found FVC to be significantly associated with lower risk of hypertension^(16,17)^, others have found the risk of hypertension to increase with higher FVC^(18)^. Some studies have also established no significant relationship between FVC and the risk of hypertension^(1,11)^. These inconsistencies in the current knowledge on the subject require more research to understand the association between FVC and the risk of hypertension, particularly in resource-constrained settings where the highest burden of hypertension is reported^(2)^. Within the Ghanaian context, there are limited studies that have explicitly investigated the association between FVC and the risk of hypertension. To narrow this knowledge gap, the study aimed to examine the association between FVC and the risk of hypertension among women in Ghana.

## Methods

### Data sources

The analysis in this study used extracted data from the most current (2014) Ghana Demographic and Health Survey (GDHS). The survey collected data from a nationally representative sample of women aged between 15 and 49 years from all the then ten administrative regions of Ghana^(19)^. Usual of nationwide representative datasets, the GDHS uses sample weights to regulate for effects of under- and over-sampling to allow for the generalisability of the results. The GDHS used a two-stage sample design. The first stage involved selecting points or clusters from an updated master sampling frame constructed from the most current Ghana Population and Housing Census^(19)^. The second stage includes a systematic sampling of households listed in each cluster. A total number of 9396 women (15–49) responded to the questionnaire; however, after dropping all missing values across the selected variables, a weighted sample of 4168 women was used in the analyses. The Demographic and Health Survey (DHS) programme granted access to the dataset. The dataset can be accessed at https://dhsprogram.com/methodology/survey/surveydisplay-491.cfm. We relied on the Strengthening the Reporting of Observational Studies in Epidemiology (STROBE)^(20)^.

### Study variables and measurements

#### Outcome variable

The primary outcome of the study was hypertension. A person with systolic blood pressure (SBP) ≥ 140 mmHg or a diastolic blood pressure (DBP) ≥ 90 mmHg was considered hypertensive and then categorised as ‘1’ and ‘0’ if otherwise. To assess the participant’s blood pressure, both systolic and diastolic values were measured using a digital oscillometric blood pressure monitoring device. These measurements were taken three times, with a 5-minute gap between each reading. Subsequently, the average of the last two measurements was calculated and recorded^(21)^.

#### Explanatory variables

Our main explanatory variables were the frequency of fruit and vegetable intake. This variable was assessed using two questions from the survey: ‘How many servings of fruit do you eat in a week?’ and ‘How many servings of vegetables do you eat in a week?’ To derive the main explanatory variable (frequency of FVC), the number of servings of fruit and vegetables was multiplied by 7 to obtain the number of times each (fruit and vegetable) was consumed in a week. To align with the recommended daily intake of a minimum of five servings of fruit and vegetables for optimal health benefits, we established the recommended number of servings per week as 35. Consequently, responses were dichotomised, assigning a value of ‘1’ to respondents consuming 35 or more servings of fruit and vegetables weekly and ‘0’ for those consuming less than 35 servings. To examine more completely the association between FVC and hypertension, multiple potential factors informed by empirical literature relating to hypertension^(8–10)^ were identified and adjusted for in the regression analysis as covariates. These factors include age, marital status, wealth, ethnicity, ecological zone, residence, occupation, religion, BMI, frequency of listening to the radio and frequency of watching television.

### Statistical analyses

Stata version 14 (StataCorp) was used as the statistical tool for all the analyses. Both descriptive and binary logistic regression were performed. Specifically, data analysis was carried out at three levels: univariate, bivariate and multivariate. The first part of the analysis is based on univariate (it examines the proportion of hypertensive and non-hypertensive) and bivariate (it explores the proportion of hypertension status by the selected explanatory variables) with its corresponding chi-square scores to test whether there was a significant association between categorical explanatory variables. In the binary logistic regression analysis, six models were fitted: Model I and II examine the association between FVC and hypertension using the SBP grouping; Model III and IV examine the association between FVC and hypertension using the DBP grouping; and finally, Model V and VI examine the association between FVC and hypertension using the combined SBP and DBP groupings. To control for potential confounding effects on the primary exposure (FVC)–outcome (hypertension) relationship, Model II, IV and VI adjusted for some socio-demographic factors that have been linked to the risk of hypertension, including age, marital status, wealth, ethnicity, ecological zone, residence, occupation, religion, BMI, frequency of listening to radio and frequency of watching television. The women’s sample weight (v005) provided in the DHS individual dataset was used in generating estimates. Due to the hierarchical nature of the GDHS and respondents being layered within survey clusters with the potential of biasing standard errors, the Huber–White technique for dealing with clustering was employed to derive robust standard errors^(22)^. To ensure that our fitted model is reliable and stable in its parameter estimation in the regression analysis, a multicollinearity test was conducted using variance inflation factor which showed a mean score of 4·87, indicating no significant multicollinearity.

## Results

### Background characteristics of respondents by hypertension status

Table 1 shows the segregated description of the explanatory variables by hypertension status in proportions. Corresponding levels of association between the explanatory variables and the outcome variable are also specified based on the Pearson chi-square test. Out of the 4168 respondents, 13·7 % were found to have hypertension. The findings indicate that there is no statistically significant link between FVC and the presence of hypertension. However, several other covariates, including age, marital status, wealth, ethnicity, ecological zone, residence, occupation, BMI and frequency of television watching, exhibited significant associations with the outcome.

**Table 1.** Background characteristics of respondents by hypertension status, 2014 GDHS (*n* 4168)

Explanatory variables	Hypertensive status			
	Non-hypertensive		Hypertensive	
	%	(*n*)	%	(*n*)
Overall	86·3	3598	13·7	570
Consumption of five servings of fruit and vegetable (X^2^, *P*-value)	(X^2^ = 0·7023, *P*-value = 0·402)			
No	85·6	1624	14·4	273
Yes	86·9	1974	13·1	297
Age (X^2,^ *P*-value)	(X^2^ = 280·1026, *P*-value < 0·001)			
< 30 years	94·8	1699	5·2	93
30–34 years	88·0	611	12·0	83
35–39 years	83·1	543	16·9	110
40–44 years	75·9	444	24·1	141
45–49 years	67·8	302	32·2	143
Marital status	(X^2^ = 105·6100, *P*-value < 0·001)			
Never married	95·4	864	4·6	42
Married	85·2	2370	14·8	411
Formerly married	75·7	364	24·3	117
Wealth index	(X^2^ = 48·9033, *P*-value < 0·001)			
Poorest	92·1	831	7·9	71
Poorer	87·8	622	12·2	87
Middle	87·1	767	12·9	113
Richer	84·8	718	15·2	128
Richest	79·5	659	20·5	170
Ethnicity	(X^2^ = 31·9154, *P*-value < 0·001)			
Akan	84·9	1514	15·1	268
Ga/Dangme	81·0	198	19·0	46
Ewe	82·5	425	17·5	90
Guan	86·2	110	13·8	18
Mole-dagbani	89·9	845	10·0	94
Grusi	90·1	167	9·9	18
Gurma	93·1	232	6·9	17
Mande	90·5	39	9·5	4
Other	83·4	67	16·6	13
Ecological zone	(X^2^ = 27·7963, *P*-value < 0·001)			
Coastal	83·6	1492	16·4	293
Forest	86·0	1087	14·0	177
Savannah	91·1	1018	8·9	100
Residence	(X^2^ = 31·6408, *P*-value < 0·001)			
Urban	83·1	1821	16·9	371
Rural	89·9	1777	10·1	199
Occupation	(X^2^ = 47·2282, *P*-value < 0·001)			
Not working	92·0	673	8·0	59
Professional/clerical	86·1	262	13·9	42
Sales/service	82·4	1346	17·6	288
Agricultural	89·4	828	10·6	98
Manual	85·5	488	14·5	83
Religion	(X^2^ = 2·6172, *P*-value = 0·454)			
Christianity	85·9	2734	14·1	449
Islam	87·3	681	12·7	99
No religion	87·3	93	12·7	13
Other	91·0	89	9·0	9
BMI	(X^2^ = 179·5163, *P*-value < 0·001)			
Underweight	92·1	132	7·9	11
Normal	92·4	1882	7·6	154
Overweight	84·6	976	15·4	177
Obese	72·8	607	27·2	227
Frequency of listening to radio	(X^2^ = 10·9800, *P*-value = 0·004)			
Not at all	90·2	597	9·8	65
Less than once	86·6	1116	13·4	173
At least once	85·0	1885	15·0	332
Frequency of watching television	(X^2^ = 15·0073, *P*-value = 0·001)			
Not at all	89·8	1023	10·2	116
Less than once	86·3	862	13·7	137
At least once	84·4	1713	15·6	317

GDHS, Ghana Demographic and Health Survey.

With respect to FVC, a similar proportion of the respondents with (13·1 %) and without (14·4 %) of the recommended servings were, respectively, hypertensive. Regarding other characteristics of the sampled respondents, among those aged 45–49 years, 32·2 % were hypertensive compared to 5·2 % in those aged less than 30 years. Concerning marital status, a significant difference was observed, with 24·3 % of formerly married women being hypertensive compared to only 4·6 % of never-married women. Similarly, 20·5 % of women with the highest wealth index were hypertensive compared to those with the poorest wealth index (7·9 %). In terms of residence type, 16·9 % of those who reside in urban areas were more hypertensive than those living in rural areas (10·1 %). Furthermore, a higher proportion of hypertensive respondents were found among those classified as overweight (15·4 %) and obese (27·2 %) compared to only 7·6 % among those with a normal weight.

Table 2 presents the results from a binary logistic regression analysis of the association between FVC and hypertension. Six models were run sequentially: Model I and II examine the association between FVC and hypertension using the SBP grouping; Model III and IV examine the association between FVC and hypertension using the DBP grouping; and finally, Model V and VI examine the association between FVC and hypertension using the combined SBP and DBP groupings. In all models, respondents’ consumption of the recommended servings of fruit and vegetables per d was not significantly associated with hypertension. In the final model (VI), age, marital status and BMI were statistically significantly associated with hypertension. Similar observations were made in the separate systolic and diastolic groupings’ models (II and VI).

**Table 2.** Binary logistic regression results of associated factors of hypertension among women

Explanatory variables	Model I		Model II		Model III		Model IV		Model V		Model VI	
	Systolic blood pressure				Diastolic blood pressure				Combined (systolic and diastolic)			
	OR	95 % CI	AOR	95 % CI	OR	95 % CI	AOR	95 % CI	OR	95 % CI	AOR	95 % CI
Consumption of five servings of fruit and vegetable												
No	Ref		Ref		Ref		Ref		Ref		Ref	
Yes	1·0	0·75, 1·24	0·9	0·69, 1·18	0·9	0·76, 1·13	0·9	0·70, 1·06	0·9	0·87, 1·37	0·8	0·70, 1·05
Age												
< 30 years			Ref				Ref				Ref	
30–34			2·3**	1·27, 4·23			1·8**	1·21, 2·61			1·7**	1·17, 2·47
35–39			5·1***	2·98, 8·76			2·5***	1·74, 3·63			2·6***	1·81, 3·68
40–44			8·2***	4·80, 14·00			3·5***	2·41, 5·04			3·6***	2·56, 5·20
45–49			15·5***	9·20, 26·22			5·2***	3·57, 7·51			6·1***	4·29, 8·73
Marital status												
Never married			Ref				Ref				Ref	
Married			1·7	0·89, 3·31			1·7*	1·14, 2·66			1·7**	1·14, 2·57
Formerly married			2·1	1·05, 4·41			2·4**	1·46, 3·92			2·3**	1·44, 3·70
Wealth index												
Poorest			Ref				Ref				Ref	
Poorer			1·3	0·74, 2·18			1·1	0·74, 1·69			1·1	0·74, 1·62
Middle			1·6	0·85, 2·93			0·9	0·59, 1·52			1·0	0·61, 1·51
Richer			1·4	0·68, 2·72			0·9	0·54, 1·58			1·0	0·57, 1·58
Richest			1·5	0·70, 3·05			1·3	0·76, 2·32			1·3	0·75, 1·20
Ethnicity												
Akan			Ref				Ref				Ref	
Ga/Dangme			0·8	0·50, 1·47			1·1	0·71, 1·64			1·0	0·68, 1·51
Ewe			1·0	0·67, 1·59			1·3	0·96, 1·85			1·2	0·91, 1·71
Guan			0·7	0·35, 1·52			0·7	0·39, 1·42			0·8	0·45, 1·46
Mole-dagbani			0·9	0·49, 1·69			0·9	0·59, 1·52			1·0	0·62, 1·53
Grusi			1·1	0·51, 2·42			1·1	0·58, 2·05			1·0	0·54, 1·86
Gurma			0·7	0·28, 1·63			1·0	0·53, 1·81			0·8	0·47, 1·56
Mande			0·2	0·04, 1·72			1·0	0·29, 3·17			0·7	0·23, 2·42
Other			0·5	0·17, 1·47			1·0	0·45, 2·11			0·8	0·35, 1·65
Ecological zone												
Coastal			Ref				Ref				Ref	
Forest			0·9	0·65, 1·25			1·0	0·75, 1·25			0·9	0·73, 1·19
Savannah			0·7	0·36, 1·21			0·8	0·50, 1·30			0·7	0·46, 1·14
Residence												
Urban			Ref				Ref				Ref	
Rural			0·6*	0·45, 0·96			0·9	0·65, 1·16			0·8	0·58, 1·04
Occupation												
Not working			Ref				Ref				Ref	
Professional/clerical			0·8	0·38, 1·87			1·1	0·68, 1·85			1·1	0·67, 1·78
Sales/service			1·3	0·78, 2·13			1·1	0·76, 1·58			1·2	0·82, 1·65
Agricultural			1·3	0·73, 2·32			1·0	0·67, 1·63			0·1	0·72, 1·66
Manual			0·4	0·09, 1·53			1·1	0·70, 1·67			1·2	0·77, 1·76
Religion												
Christianity			Ref				Ref				Ref	
Islam			1·6	1·03, 2·42			1·0	0·74, 1·48			1·1	0·83, 1·60
No religion			1·3	0·60, 3·04			1·0	0·50, 2·06			1·0	0·54, 2·05
Other			0·4	0·09, 1·54			0·7	0·33, 1·63			0·8	0·36, 1·60
BMI												
Normal			Ref				Ref				Ref	
Underweight			0·8	0·35, 2·09			1·3	0·73, 2·36			1·2	0·66, 2·08
Overweight			1·3	0·94, 1·90			1·7***	1·28, 2·20			1·6***	1·24, 2·07
Obese			1·7**	1·16, 2·43			2·7***	2·00, 3·58			2·4***	1·82, 3·20
Frequency of listening to radio												
Not at all			Ref				Ref				Ref	
Less than once			0·9	0·61, 1·47			1·4	0·96, 2·03			1·2	0·88, 1·75
At least once			1·2	0·77, 1·78			1·4	0·97, 2·01			1·3	0·94, 1·81
Frequency of watching television												
Not at all			Ref				Ref				Ref	
Less than once			0·9	0·60, 1·45			1·2	0·87, 1·74			1·0	0·72, 1·39
At least once			1·01	0·66, 1·54			1·3	0·97, 1·90			1·1	0·84, 1·59
Constant	0·1***	0·06, 0·08	0·0***	0·00, 0·02	0·1***	0·11, 0·14	0·0***	0·01, 0·03	0·1***	0·13,0·16	0·0***	0·01,0·04
Model fitness												
Prob > chi2	0·7945		< 0·001		0·4489		< 0·001		0·4021		< 0·001	
AIC	1966·334		1687·509		2860·564		2563·01		3089·136		2744·679	
Pseudo R^2^	0·0000		0·1778		0·0002		0·1288		0·0002		0·1345	
* n*	4168		4168		4168		4168		4168		4168	

Ref, reference category; AOR, adjusted OR.

* *P* < 0·05.

** *P* < 0·01.

*** *P* < 0·001.

Based on the final model (VI), the risk of developing hypertension was found to be associated with increasing age. For instance, a higher likelihood of being hypertensive was observed among women within their late reproductive age (45–49 years) (adjusted OR (AOR) = 6·1; CI = 4·29, 8·73) compared to those aged less than 30 years. Additionally, the likelihood of being hypertensive was higher among married (AOR = 1·7; CI = 1·14, 2·57) and formerly married women (AOR = 2·3; CI = 1·44, 3·70) than their counterparts who were never married. Notably, compared to women with normal body weight, those who were overweight (AOR = 1·6, CI = 1·24, 2·07) and obese (AOR = 2·4, CI = 1·82, 3·20) were more likely to be hypertensive.

## Discussion

This study examined the association between FVC and the risk of hypertension among women in Ghana using nationally representative data. We found no significant association between FVC and hypertension risk. The result from the study is inconsistent with previous studies that have found a significant association between FVC and the risk of hypertension^(16,17)^. However, it is corroborated by some studies that found no significant association between FVC and the risk of hypertension^(1,11,23)^. The observed non-significance of FVC in relation to hypertension risk may reflect the effects of strong confounders, including their level of physical activity, Na intake and cooking practices which could not be accounted for in this study. For instance, Miglio et al.^(24)^ assert that cooking practices such as boiling and frying vegetables significantly reduce their nutritional quality and antioxidant compounds necessary for stimulating biological mechanisms that support reduced risk of hypertension. It is also possible that the no significant association observed in this study may be evidence of the point that it might require consistent long-term consumption of fruits and vegetables to exhibit a significant effect on hypertension risk. Longitudinal studies would be needed in the future to investigate the dose–response relationship between FVC and hypertension risk in Ghana.

Increasing age was associated with a higher likelihood of being hypertensive, with those in the late reproductive age (45–49 years) being 6·1 times more likely to have hypertension when compared to those aged less than 30 years. This is consistent with previous studies from Dubai^(25)^, China^(26)^, and SSA^(27)^ that have found a positive association between increasing age and higher risk of hypertension. Probably, this result can be explained by biological changes that occur with ageing such as ‘structural changes in the arteries and especially with large artery stiffness’^(28)^.

Consistent with prior literature^(25,29)^, the study found higher odds of hypertension among women who were overweight or obese. Maintaining a normal weight is essential to prevent the occurrence of impaired glucose tolerance, increased insulin levels and concomitant reductions in insulin sensitivity which often tend to be biological pathways for higher risk to high blood pressure among obese individuals^(30)^. Therefore, being overweight or obese exacerbates insulin resistance and sympathetic nervous system activation which are known biological pathways for the development of hypertension^(31,32)^.

The study also shows a significant association between marital status and hypertension risk, with formerly married women reporting a higher likelihood of being hypertensive. Our observation is corroborated by Nyarko^(33)^ whose study found a significantly higher risk of hypertension among formerly married women than among those who had never married. One study has shown that divorced and widowed women often experience loneliness and a reduced sense of life satisfaction^(34)^. Such psychological stressors influence formerly married women to engage in unhealthy lifestyles such as smoking, binge eating, alcohol consumption and less physical activity^(35)^, hence, exacerbating the risk of hypertension.

### Strengths and limitations

One of the strengths of our study is the use of a large sample size that is nationally representative. Also, we applied the appropriate statistical analysis to arrive at our findings. These are clearly stated in our methods to ensure the replicability of our study to similar contexts. Nonetheless, the use of the DHS does not permit us to make causal inferences due to the cross-sectional design of the study. Since we relied on secondary data, we were unable to account for how other important factors like cultural norms, Na intake, cooking practices, and level of physical activity influence the association between FVC and the risk of hypertension. For instance, both Na consumption and physical activity are recognised as Level 1 factors in moderating blood pressure. The lack of data on these crucial variables limits the comprehensive understanding of potential contributors to blood pressure variations within the study cohort. Future research endeavours should consider incorporating thorough assessments of Na intake and physical activity levels to enhance the robustness of findings and provide a more nuanced insight into the factors influencing blood pressure dynamics. FVC was self-reported; hence, there is a likelihood of recall and social desirability bias. While the study relied on a nationally representative dataset, this data is 10 years old. The implication is that some of the findings, for instance, relating to ethnic differences may not reflect the current situation. Hence, the interpretation of our findings should be made with caution.

## Conclusion

The study concludes that there is no significant association between FVC and hypertension risk among women in Ghana. While this study did not find a significant association between FVC and hypertension risk among women in Ghana, it underscores the point that other multifaceted factors influence hypertension risk. As such, public health campaigns should emphasise a balanced and holistic approach to promoting cardiovascular health, including factors beyond FVC. The findings also highlight the need to target high-risk populations (i.e. older women, formerly married women, and overweight or obese women) with hypertension prevention education and related interventions.
